# Androgen receptor: a potential therapeutic target for glioblastoma

**DOI:** 10.18632/oncotarget.25007

**Published:** 2018-04-13

**Authors:** Nomi Zalcman, Tamar Canello, Haim Ovadia, Hanna Charbit, Bracha Zelikovitch, Anat Mordechai, Yakov Fellig, Stav Rabani, Tal Shahar, Alexander Lossos, Iris Lavon

**Affiliations:** ^1^ Leslie and Michael Gaffin Center for Neuro-Oncology, Hadassah-Hebrew University Medical Center, Jerusalem, Israel; ^2^ Department of Neurology, Agnes Ginges Center for Human Neurogenetics, Hadassah-Hebrew University Medical Center, Jerusalem, Israel; ^3^ Department of Oncology, Hadassah-Hebrew University Medical Center, Jerusalem, Israel; ^4^ Department of Pathology, Hadassah-Hebrew University Medical Center, Jerusalem, Israel; ^5^ The Laboratory for Molecular Neuro-Oncology, Department of Neurosurgery, Tel Aviv Medical Center, Tel Aviv, Israel; ^6^ Department of Neurosurgery, Shaare Zedek Medical Center, Jerusalem, Israel

**Keywords:** androgen receptor (AR), gliomas, AR variant 7 (AR3), AR antagonist, glioblastoma (GBM)

## Abstract

The median survival time of patients with glioblastoma is still poor (14.6 month), partly due to a lack of effective treatment.

We have observed that androgen receptor (AR) is amplified in glioblastomas at the DNA, RNA and protein levels. The AR gene was amplified in 27% of glioblastoma specimens from men (n=22) and of 38.2% from women (n=21). AR-RNA was overexpressed (>2.5 fold) in 93% (n=30), and AR-protein was induced (>two fold) in 56% of the glioblastomas samples (n=16). Thirty percent of the glioblastomas (n=21) also expressed a constitutively active AR-splice-variant (AR-V7/AR3) lacking the Ligand-Binding-Domain. Following these findings, we examined the effect of pharmacological inhibition of androgen receptor *in vitro* and *in vivo*, as well as of genetic silencing of the receptor in glioblastoma cell lines. AR antagonists, induced concentration-dependent death in three glioblastoma cell lines, as well as in two glioma initiating cell lines. Silencing of AR expression by siRNA induced cell death in the three tested glioblastoma cell lines. Enzalutamide given orally to nude mice bearing subcutaneous human glioma xenografts resulted in a 72% reduction in tumor volume (p=0.0027).

The presence of AR-V7/AR3 in glioblastoma, together with the present data showing that genetic silencing of the full length AR in cell lines and pharmacological inhibition of AR, induce GBM cell death *in vivo* and *in vitro*, point to the important role of AR in GBM survival and render a potential therapeutic target for this devastating disease.

## INTRODUCTION

Glioblastoma (GBM) is the most common and the most aggressive primary brain tumor, with a very poor prognosis. The most common genetic aberrations associated with malignant glioma are amplification or activating mutations of the epidermal growth factor receptor (EGFR) or both [1–3]. Activation of EGFR results in a downstream phosphoinositol 3 kinase (PI3 kinase)/Akt cascade, facilitating cell survival, proliferation, and migration, and thus is crucial to tumorigenesis.

The association between sex steroid receptors and brain tumors was first described in 1983 [4], but in contrast to the well-established oncogenic role played by androgen receptor (AR) in prostate cancer (reviewed in [5]) and the growing evidence of its role in breast cancer (reviewed in [6]), the expression and significance of AR in GBM is controversial and poorly studied.

The AR gene is mapped to Xq12. It functions as a steroid-hormone-activated-transcription-factor and is composed of the N-Terminal-Regulatory-Domain, the DNA-Binding-Domain and the Ligand-Binding-Domain (LBD). In the presence of ligand, the ligand-binding domain is released from heat shock protein and AR is translocated into the nucleus, where it binds to the androgen-response-element in the promoter and stimulates transcription of androgen- responsive genes [7]. It has been shown in prostate and breast cancers that activation of AR can be achieved also by ligand-independent signaling through crosstalk with other molecular signaling pathways, as a consequence of activation of the downstream PI3K/AKT/mTOR. Such pathways include receptor tyrosine kinases (RTKs), such as the insulin-like growth factor keratinocyte growth factor (KGF), EGFR and Human Epidermal Growth Factor Receptor 2 (HER2) [8–11]. These AR-independent pathways can promote cancer cell survival and growth (reviewed in [7]) and appear to be a major androgen-independent driver of AR-regulated gene expression in castration-resistant prostate cancer.

AR splice variants lacking the LBD, such as AR-V7/AR3, which arise primarily through exon skipping and cryptic exon inclusion, are activated by a ligand-independent mechanism such as the ones mentioned above. [8]. Although AR-regulated signaling pathways are well established in prostate cancer, the involvement of AR in GBM and the potential AR-dependent or independent regulated signaling pathway in GBM is not known.

AR signaling inhibitors such as enzalutamide target the AR signaling pathway at three key stages: by blocking binding of androgens to AR, by inhibiting nuclear translocation of activated AR, and by impairing binding of activated AR to DNA [9].

The findings from our laboratory, some of which were previously presented [10], show amplification and overexpression of AR in GBM. This led to the present investigation of the effect of pharmacological inhibition of AR *in vitro* and in the subcutaneous *in vivo* model, as well as the effect of genetic silencing of the receptor in GBM cell lines.

## RESULTS

### Variation in AR DNA copy number and in RNA and protein expression levels in GBM

Focal CNV analysis of the AR chromosomal region with Droplet Digital™ PCR (ddPCR™) showed AR amplification in 27% of GBM specimens from men (n=22) and 38.2% of women (n=21) (Table 1). Amplification of the AR gene was observed (two copies) also in the T98G cell line, derived from the GBM of a male patient, but not in U87MG or A172. The analysis was performed following the observation, made according to an OncoScan FFPE array, of LOH at the chromosomal locus containing the AR gene, accompanied by amplification of the other allele in 4/5 GBM specimens from women [10] (Supplementary Figure 1). To determine whether this AR gene variation influences AR expression, AR-RNA and protein expression were studied.

**Table 1:** AR copy number variation (CNV) in GBM

Men (n=22)			Women (n=21)		
AR Copy number	No.	%	AR Copy number	No.	%
1	16	72.7%	1	6	28.5%
2	5	22.8%	2	7	33.3%
3	1	4.5%	3	8	38.2%

DNA was extracted from tumors of 22 men and 21 women and CNV was studied with ddPCR.

**Figure 1:**
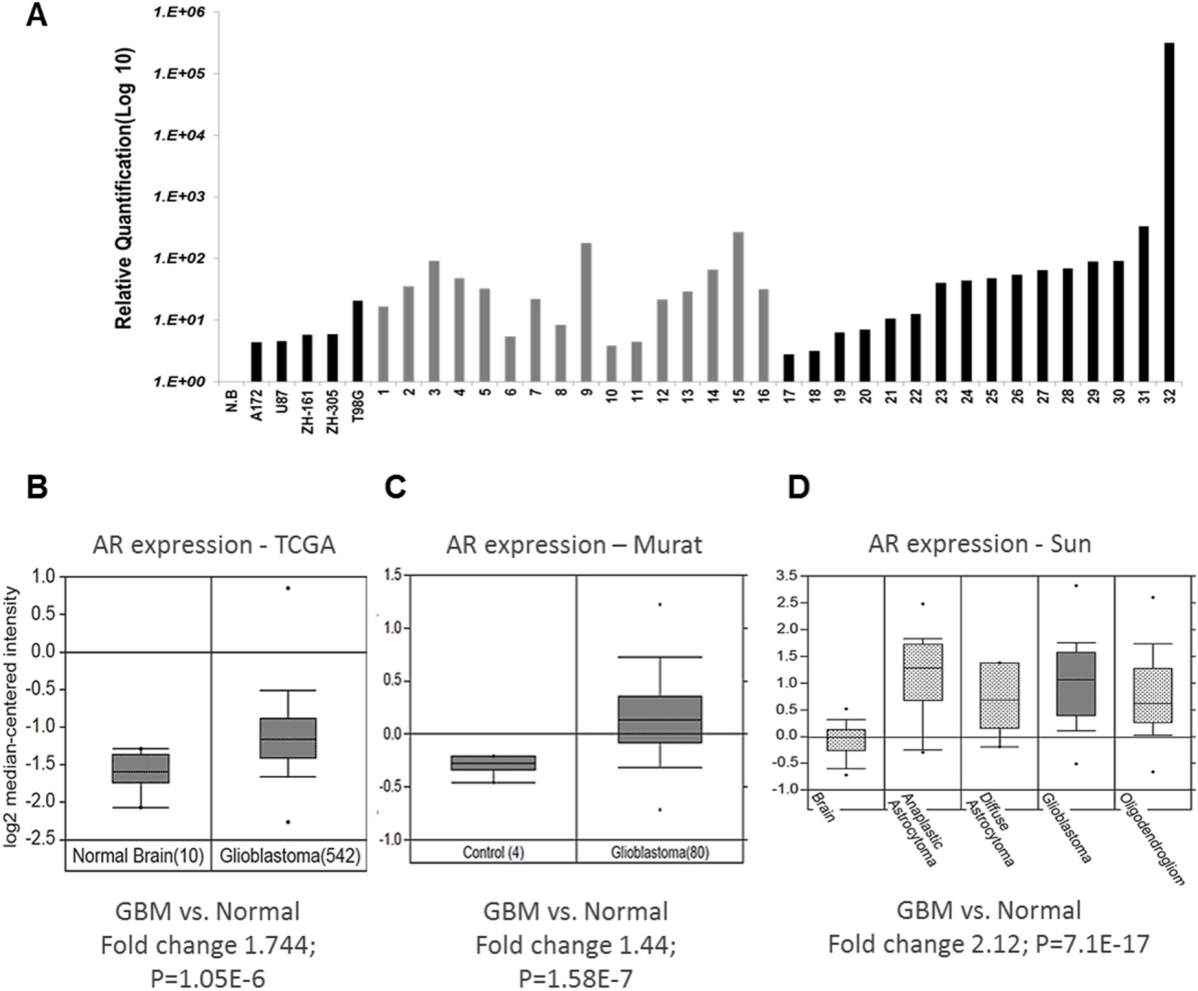
AR RNA expression in GBM tissue samples **(A)** Quantitative real-time RT PCR of RNA extracted from GBM samples of both male and female patients shows significant (2.76- to 315-fold) induction of AR-RNA in 93% of the samples. Relative AR mRNA quantification was compared with that of a commercial RNA mixture of 23 normal brains (NB); following normalization to HPRT and TBP1 (The Y-axis is on a log_10_ scale) in 30 GBM tumor samples and three GBM cell lines and two GIC cell lines (X-axis). **(B-D)** AR RNA expression in tumors compared with that in normal tissue analyzed from several datasets with Oncomine™ (B) GBM AR RNA expression of the TCGA database; (C) GBM AR RNA expression of the Murat cohort; (D) glioma AR RNA expression of the Sun cohort.

Quantitative-PCR (qPCR) showed that AR-RNA was overexpressed (2.76-315,984 fold) in 93% of 30 glioblastoma samples of both men and women patients (Figure 1A; Table 2). Further analysis of AR RNA amplification from several datasets, including the TCGA dataset, with Oncomine™ software (Compendia Bioscience, Ann Arbor, MI, USA), validated the RNA results (Figure 1B-1C) and showed that AR-RNA is overexpressed also in low-grade gliomas (Figure 1D). Analysis of prostate cancer datasets revealed that the AR RNA fold change in prostate cancer is similar to the present findings in GBM (Supplementary Figure 2A, 2B).

**Table 2:** Characteristics of 32 patients with glioblastoma

ID (Figure 1A, 1E)	Sex	Age	Survival (month)	MGMT Methylation	Admission ECOG score
**1**	M	54	5.6	UM	1
**2**	F	48	8.1	UM	1
**3**	M	61	4	ME	1
**4**	M	30	83	ME	0
**5**	F	58	31.2	UM	1
**6**	F	66	27.6	ME	0
**7**	F	37	52.7	ME	0
**8**	F	67	27.1	UM	0
**9**	M	63	7.6	ME	0
**10**	F	72	6.5	UM	1
**11**	F	68	71.3	ME	0
**12**	F	30	34.1	UM	1
**13**	F	55	21.9	UM	0
**14**	M	34	66.5	UM	0
**15**	M	50	4.2	UM	1
**16**	M	71	4.3	UM	0
**17**	M	60	3.5	UM	1
**18**	F	59	29.6	UM	1
**19**	M	62	28.2	UM	1
**20**	F	74	5.5	UM	1
**21**	F	68	17.7	UM	0
**22**	F	70	4	ME	1
**23**	M	55	7.8	ME	2
**24**	M	57	83	ME	0
**25**	M	63	111.4	UM	1
**26**	M	59	30.8	UM	1
**27**	M	62	9.3	UM	1
**28**	F	68	63.1	ME	0
**29**	F	54	33.3	ME	0
**30**	M	62	4.1	ME	1
**31**	F	63	73.9	ME	0
**32**	M	68	8.9	UM	1

Abbreviations: F, female; M, Male; ME, methylated; UM, unmethylated; MGMT, O6-methylguanine methyltransferase ECOG, the Eastern Cooperative Oncology Group Performance Scale.

IDH1 status: Patients (8) and (22) carry the IDH1 R132H mutation, in the rest of the patients IDH is intact.

Detailed correlation analysis between AR expression and clinical parameters was adapted from the analysis performed on 24,519 genes by the Broad Institute TCGA Genome Data Analysis Center in 519 GBM tumors [11]. The analysis showed a significant positive correlation between AR expression and age (R=0.1105; P=0.01132; Q = 0.0765) and a significant negative correlation with the Karnofsky performance score (R= -0.0978; P= 0.05173; Q= 0.324). No correlation was found between AR expression and survival, gender, radiotherapy, race or ethnicity (Table 3).

**Table 3:** Correlation between AR expression and clinical features

Clinical features	Statistical test and results
**Days to death or last follow up**	Logrank P=0.787 Q = 0.920
**Years to birth**	Spearman Correlation R=0.1105 P=**0.01132** Q = **0.0765**
**Gender**	Wilcoxon test P = 0.8432 Q= 0.971
**Radiation therapy**	Wilcoxon test P = 0.895 Q= 0.98
**Karnofsky performance score**	Spearman Correlation R= -0.0978 P= **0.05173** Q= 0.324
**Race**	Kruskal wallis P =0.2541 Q=1
**Ethnicity**	Wilcoxon test P = 0.351 Q= 0.952

Significance (bold) when P value < 0.05 and Q value (False Discovery Rate) < 0.3.

A table drawn from the analysis made by the Broad Institute TCGA Genome Data Analysis Center [11] showing the results of the association between the clinical features and AR expression across 519 tumor samples according to the statistical methods used.

**Figure 2:**
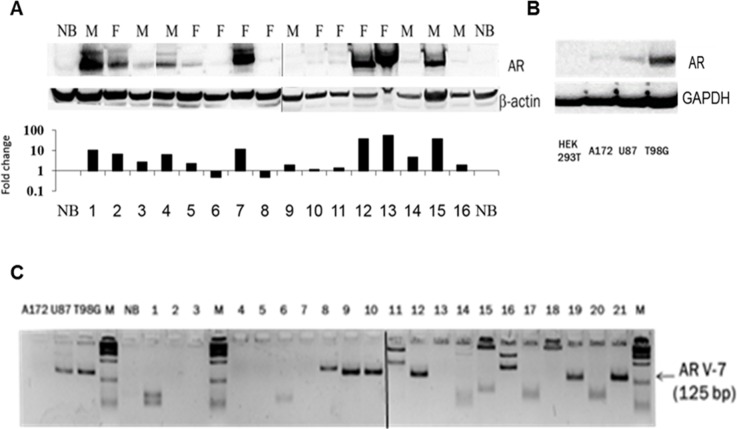
AR protein expression and the presence of the ligand-independent AR splice variant 7 (AR3) in GBM samples **(A)** Western blot analysis by sequential probing with polyclonal antibody against AR (N20) or anti-β-Actin (AC-74) of 16 GBM samples (these samples were also analyzed for AR-RNA expression and are marked in grey in Figure 1A) of Females (F) and male (M) and one normal brain. The figure is composed of two gels that were run simultaneously. Protein fold change (the Y-axis is on a log_10_ scale) of each tumor sample compared with that of normal brain was calculated according to band densitometry analysis with alphaview software, following normalization to β-Actin (lower panel). **(B)** Western blot analysis, with sequential probing with polyclonal antibody against AR (N20) or monoclonal anti- GAPDH (0411) of HEK 293, A172, U87MG and T98G cell lines. **(C)** AR-V7/AR3 was analyzed by quantitative PCR of 21 GBM specimens, a commercial mixture of total RNA from 23 normal brains (NB) and 3 glioma cell lines, as indicated. The resulting 125-bp fragments, together with a 100-bp DNA ladder (M) were electrophoresed on 3.5% metaphor and visualized with ethidium bromide. The figure is composed of two gels that were run together.

Western blot analysis revealed AR protein induction (2-106-fold) in 56% of the glioblastomas samples (n=16) of both men and women patients (Figure 2A; Table 2), and in the three GBM cell lines tested (2B).

### AR-V7/AR3, which lacks the ligand-binding domain, occurs in GBM

The AR-V7/AR3, known to be upregulated only in tumors and activated via a ligand-independent mechanism, was found in 30% of the GBM surgical specimens, in addition to the wild type AR (n=21) (Figure 2C).

### Antagonism of AR in glioma cell lines induces dose-dependent cell death

**Figure 3:**
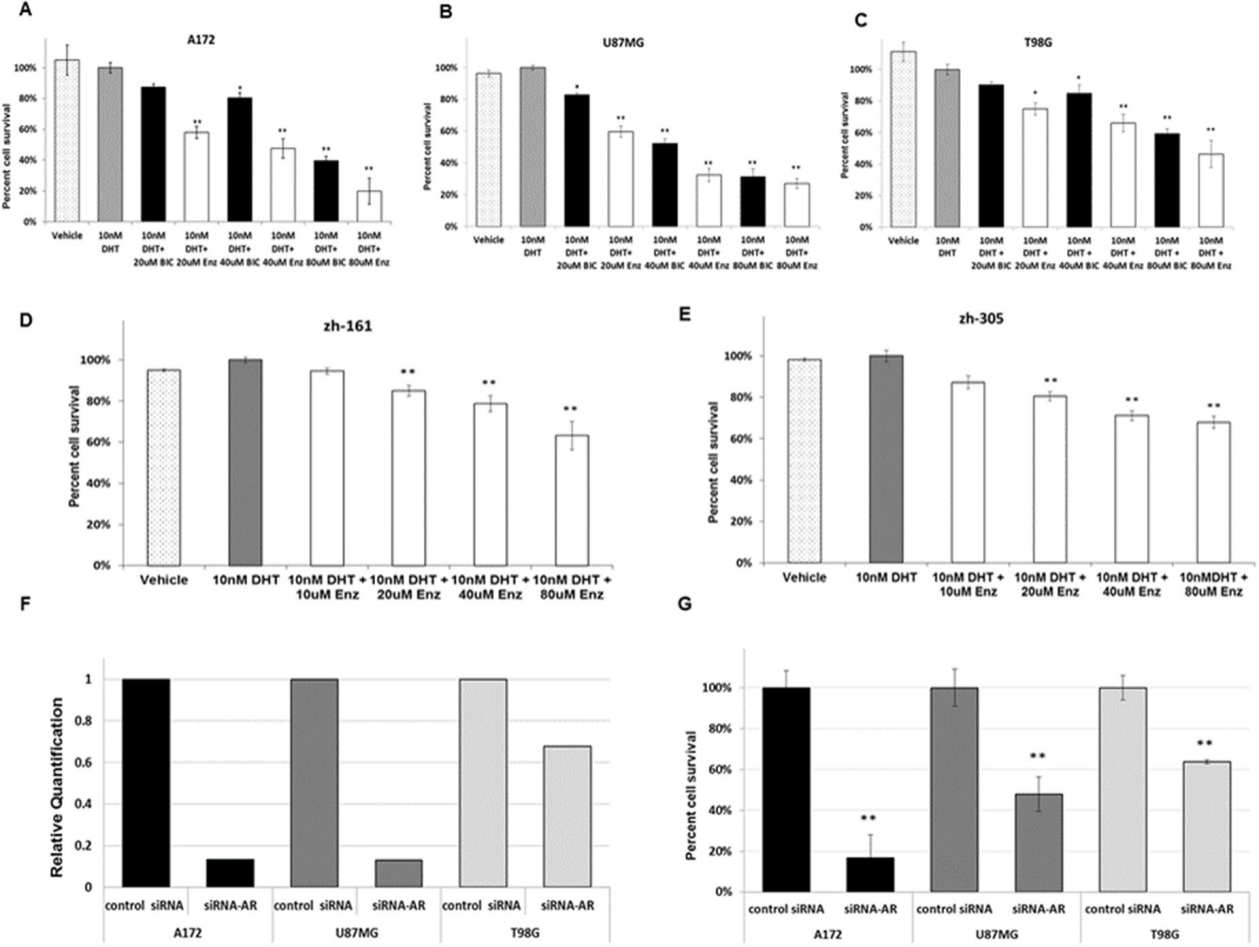
Pharmacological inhibition and silencing of AR in glioma cell lines **(A-C)** GBM cell lines were maintained in culture medium supplemented with androgens (as well as other steroids)-stripped FBS. **(D, E)** GIC cell lines were treated with neurobasal medium without FBS. (A-E) both GBM cell lines and GIC cell lines were treated with vehicle (0.15% ethanol) (white bars with black dots) or with a physiological dose (10nm) of DHT alone (gray bars) or in combination with the indicated doses of bicalutamide (BIC, black bars) or enzalutamide (ENZ, white bars) (X-axis) for 72 hrs. Cell viability was determined as described in Methods and is expressed as the percentage of viable cells following treatment with DHT (Y-axis). (A) A172 cell line; (B) U87MG cell line; (C) T98G cell line; (D) ZH-161cell line; (E) ZH-305 cell line. **(F-G)** Cells were transfected with 1.2 nM of non-targeted siRNA (control siRNA) or siRNAs targeting human AR. (F) RNA interference determined by Quantitative real-time RT PCR 24 hrs later. (G) Cell viability determined with the crystal violet assay 72h later. All experiments were repeated at least three times. The results of the viability experiments are presented as the mean ± SD ^*^P<0.05, ^**^P<0.01 versus control group.

The finding of AR overexpression in GBM in the present study and based on the documented benefit of AR inhibitors in prostate cancer, the effect of AR inhibitors bicalutamide and enzalutamide on the survival of glioma cell lines A172, U87MG and T98G was explored. Prostate carcinoma cell line PC3, which does not express AR [12], served as negative control. Both inhibitors induced significant (p<0.05 and p<0.01, as indicated) dose-dependent cell death in all glioma cell lines, enzalutamide showing the higher efficacy (Figure 3A-3C). No effect was seen in the PC3 line (Supplementary Figure 3A). To standardize the experiments and avoid the influence of Fetal Bovine Serum (FBS)-derived steroid hormone, the cell medium was supplemented with charcoal/dextran-treated (stripped) FBS and a physiological dose of DHT (10nM). The DHT dose had no effect on the proliferation of the GBM cell lines (Figure 3). Treatment of the cells with enzalutamide in cell medium supplemented with full serum, without the addition of DHT, yielded similar results (Supplementary Figure 3B, 3C).

In view of the results obtained with the standard cell lines A172, U87MG and T98G, the efficacy of enzalutamide was tested also on two glioma-initiating cell (GIC) lines, ZH-161 and ZH-305 [13, 14]. Both GIC lines, like the T98G cell line, expressed high levels of full length AR (Figure 1A) and even higher levels of AR-V7/AR3 (1.79 and 3.5-fold respectively compared with T98G). Treatment of the lines with enzalutamide showed low but significant efficacy (Figure 3D, 3E).

### Silencing of AR with siRNA in glioma cell lines induces cell death

To test whether AR has a specific effect on GBM cell viability, the three GBM cell lines were transfected with siRNA targeted to the full length AR or with non-targeted siRNA. Real time RT-PCR, confirmed a reduction in AR transcription in cells transfected with siRNA targeted to the full length AR compared with that in cells transfected with control siRNA. In all the three cell lines there was a correlation between tumor cell death and the extent of reduction of AR expression (Figure 3F, 3G).

### AR antagonist induces apoptotic cell death

**Figure 4:**
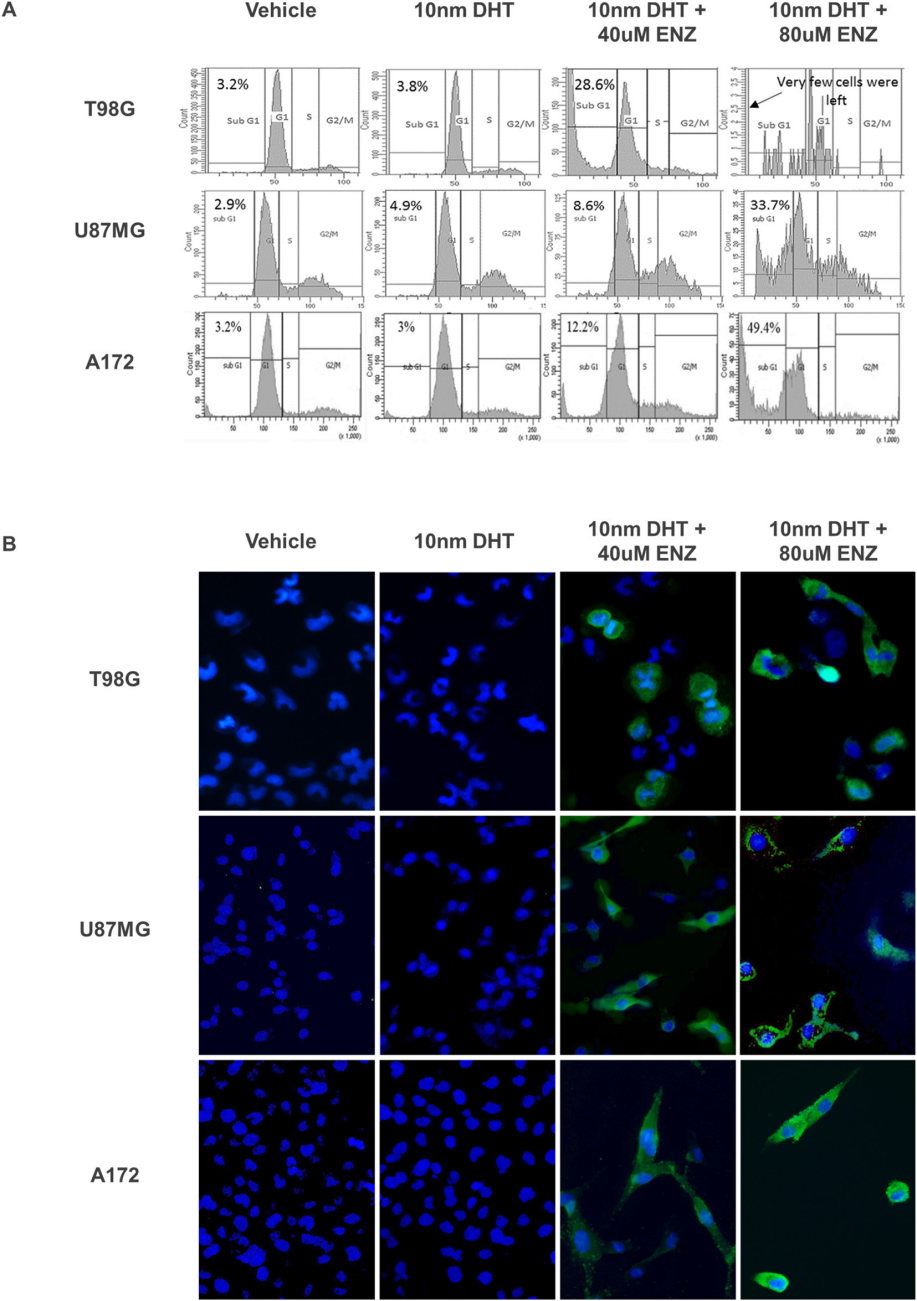
Analysis of PI labeling and cleaved caspase 3 in the A172 U87MG and T98G cell lines after enzalutamide treatment Glioma cell lines were treated with vehicle (0.15% ethanol), 10nm DHT with or without 40 or 80μM enzalutamide, as indicated and subjected to PI labeling **(A)** or to cleaved caspase 3 immunofluorescence **(B)**. (A) DNA content histogram following cell cycle analysis with propidium iodide (PI). PI fluorescence intensity was captured with flow cytometry via the FL2 channel and 488nM laser excitation. The percentage of cells at sub-G1 is indicated for each treatment. Enzalutamide sensitive cell lines A172 and U87 were analyzed after 48 hrs, T98G - after 72 hrs. (B) 72 hrs after treatment with enzalutamide the cells were fixed and incubated with primary antibody for Cleaved Caspase-3 (Asp175) and Alexa Fluor 488 conjugate secondary antibody (green). Before visualization the cell nuclei were stained with DAPI (blue). The cells were captured with a EVOS® Digital Microscope at x 200 magnification.

Cell cycle analysis of the three glioma cell lines treated with enzalutamide revealed a dose-dependent number of cells in the sub-G1 phase, suggesting that apoptosis was one of the mechanisms responsible for cell death (Figure 4A). The finding was further validated by immunohistochemical staining of the enzalutamide-treated cells with anti-cleaved caspase 3 (Figure 4B).

### AR antagonist enzalutamide significantly reduces the growth of human glioblastoma in a xenograft mouse model

**Figure 5:**
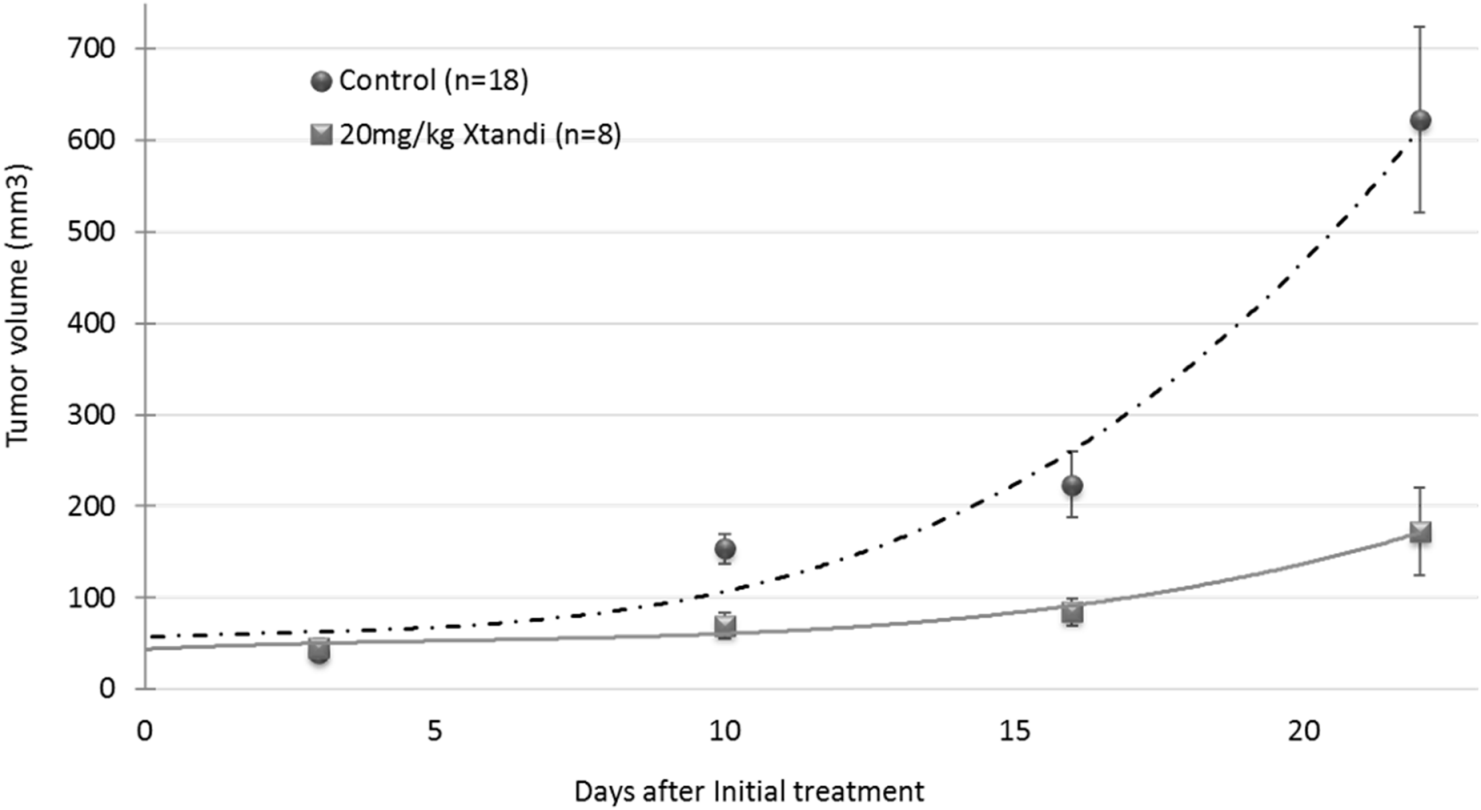
*In vivo* efficacy of the androgen receptor antagonist enzalutamide (XTANDI) in U87MG human glioblastoma xenografts A total 5×10^6^ tumor cells were inoculated subcutaneously into athymic nude mice, which were randomly assigned to vehicle (n=18) and XTANDI (n=8) treated groups. Treatment started on day 7 when the tumors had grown to an approximate 50-mm3volume. The mice were gavaged orally every second day with 20/mg/kg XTANDI or vehicle (220mg/kg caprylocaproyl polyoxylglycerides CH in saline). Each point, representing median tumor volume ± SEM, is shown with polynomial curve fitting.

The efficacy of enzalutamide was studied *in vivo* in subcutaneous xenografts of U87MG human glioblastoma. On day 7 when the tumors reached an average volume of about 50 mm^3^, the mice were randomly assigned to two treatment groups, based on caliper measurements. All the mice were treated three times weekly by oral gavage of 20 mg/kg XTANDI® (enzalutamide) or vehicle. The study was terminated on day 31 post tumor inoculation when the tumor volume of half of the mice in the vehicle group reached 800–1000 mm^3^. At the endpoint of the experiment, there was a 72% reduction in tumor volume in the group treated with XTANDI vs that in mice treated with vehicle (p=0.0027) (Figure 5)

## DISCUSSION

The results of this study show for the first time AR amplification at the DNA, RNA and protein levels in GBM samples from both men and women. The AR-RNA expression findings were validated in 703 glioblastomas by analysis of several datasets, including the TCGA. Bao et al.[15] confirmed some of our previous results [10] regarding AR protein expression. No correlation was found between the expression of AR RNA and AR protein. This discrepancy may be related to differential post-transcriptional regulatory mechanisms involving the AR gene.

The significant negative correlation of AR expression with the Karnofsky performance score obtained from the detailed correlation between AR expression and clinical parameters according to the TCGA dataset, may suggest that there is a correlation between AR expression and tumor aggressiveness. The finding that there was no correlation between AR expression and survival could be attributed to the non-standardized background of the clinical dataset. The absent correlation between AR expression and gender supports our findings that AR overexpression occurs in tumors from both men and women.

AR copy number variation is probably not the sole mechanism responsible for AR RNA and protein overexpression. This may be concluded from the finding that AR DNA amplification was found only in the T98G cell line (1/3 lines), whereas AR RNA and protein overexpression was found in all cell lines (with higher overexpression in the T98G cell line). Furthermore, only about 1/3 of the specimens showed AR DNA amplification, whereas 93% of the patients demonstrated AR RNA overexpression and 56% showed AR protein overexpression. A possible other mechanism may include activation of the enhancer in the AR second intron contributing to increased AR expression at low androgen levels, as in castration-resistant prostate cancer [16].

In the present investigation, pharmacological inhibition of AR with AR antagonists in three glioma cell lines, as well as in two GICs, induced dose concentration-dependent death. Cell cycle analysis of the AR antagonist- treated cells, as well as staining with anti- cleaved caspase 3, suggest that the mechanism responsible for cell death is at least in part, apoptosis. Genetic silencing of AR expression by siRNA resulted in reduced GBM cell viability. These finding validate the results of the pharmacological AR inhibition and indicate that AR has a specific role in GBM cell survival.

In addition, we now demonstrate that 30% of GBM samples expressed constitutively-activated AR-V7/AR3 lacking the LBD. This variant is a major androgen-independent driver of AR-regulated gene expression in advanced prostate cancer [8]. The existence of such a variant in GBM, together with the finding that silencing of the full length AR induces GBM cell death, might point to the important role played by AR in GBM growth.

The AR-V7/AR3 variant was not detected by Bao et al. [15] in U87MG or U251 glioma cell lines, probably because of differences in the sensitivity of the methods used. In the present study, the standard industrial qPCR thermocycling protocol of 40 amplification cycles was used and AR-V7/AR3 was detected in U87MG at cycle 34, whereas Bao et al., used a semi-quantitative PCR analysis and failed to detect AR-V7/AR3 at cycle 27. The group did not check the presence of AR-V7/AR3 in glioma specimens.

T98G, as well as the GIC cell lines, show high expression of the full length AR and of the constitutive active AR variant. These cell lines showed reduced but still significant susceptibility to AR inhibitors. This residual sensitivity to AR inhibitors could be related to the fact that the mechanism of action of inhibitors such as enzalutamide [9] include, apart from blocking the binding of androgens to AR, inhibition of the nuclear translocation of the activated AR, and impaired binding of activated AR to DNA. Thus, such inhibitors might be active also against ligand-independent AR activation. Moreover, whether AR variants drive therapeutic resistance remains unresolved. Some studies have demonstrated that knockdown of AR-V in cell lines that endogenously express high levels of the variant, restores their sensitivity to anti-androgens [17, 18]. In contrast, other studies in prostate carcinoma indicate that overexpression of AR-V7 did not impart resistance to enzalutamide [19]. Additionally prostate cancer cell line derived from the Hi-Myc mouse, which expressed AR-V, is sensitive to enzalutamide [20].

It has been shown in prostate and breast cancers that AR activation might be achieved through ligand-independent mechanisms. These may include increased coactivator expression and activation of several kinases that may directly or indirectly activate AR. [16]. This is the case also in a triple-negative breast cancer, in which AR appears to drive tumor progression. Likewise, other signaling pathways, such as ERK, can enhance AR transcriptional activity through phosphorylation of AR and its co-regulators (for review see [17]). Such mechanisms may be involved in AR activation in GBMs that are usually exposed to minor levels of AR natural ligands (e.g., testosterone and DHT), especially in women. The existence of these mechanisms in brain tumors warrants further investigation.

Since EGFR amplification or activating mutations [1–3] are the most common genetic aberrations associated with malignant glioma, a combination therapy with anti-AR agent and EGFR inhibitor might have a beneficial effect in GBM especially in tumors that carry the AR variant and are known to be activated via signal transduction pathways, such as the EGFR pathway [17–19]. This hypothesis remains to be investigated *in vitro* and *in vivo*.

With the aim of translating the *in vitro* results to the clinic, an attempt was now made to find *in vivo* proof of concept. The *in vivo* experiment described here shows that the AR antagonist enzalutamide efficiently reduces the growth of human glioblastoma. This was recently also demonstrated by Bao et al. [15].

Androgen receptor signaling pathways in gliomas remain largly unknown. A recent study demonstrated that treatment of U87MG with 500 ng/ml DHT decreased cell apoptosis driven by treatment with 10 ng/ml TGFβ1 [21]. The authors concluded that AR signaling might promote tumorigenesis of GBM through TGFβ1 [21]. Another recent publication proposes AR promotion of glioma progression by suppressing SVIP and p53 [15].

Molecular analysis of tumors is becoming more detailed, allowing therapy tailored to individual tumor types. Thus, it is becoming clear that no single treatment will be the panacea for gliomas. Therefore, if the multi-faceted treatment approach is to be successful, it would have to utilize as many targets for therapy as logistically possible. AR is an excellent target for therapy, as it has been shown historically to be an effective target for prostate cancer treatment. In contrast to prostate cancer, efficient penetration of the AR antagonist through the blood-brain-barrier is obligatory for the treatment of brain tumors. Thus, screening of the blood-brain-barrier penetration of an already existing AR antagonist is still warranted in order to test the efficacy of AR antagonist in an intracranial *in vivo* model. Furthermore, a clear and comprehensive understanding of the mechanism of AR signaling in GBM has yet to be revealed. Following the detection of AR signaling in GBM, the gene expression biomarker of the AR response could be used to assess the responsiveness to AR inhibition *in vivo*. Hopefully, the results of the present study, in concert with continued laboratory efforts, may lead to a new approach for the treatment of human glioblastoma.

## MATERIALS AND METHODS

### Patients and tumors

A DNA study was performed on 48 paraffin-embedded tumors of adult men and women suffering from GBM, (the exact number of samples used for each study is indicated), who were operated at the Hadassah Hebrew University Medical Center. The study was approved by the local institutional research ethics committee, and all patients signed written consent forms (IRB-0099-09-HMO).

An expression study was performed on samples from 32 newly diagnosed primary GBM adult men and women patients who were operated at the Tel Aviv Medical Center (Table 2). Patients signed their written informed consent according to an institutional review board-approved protocol (TLV 0307-09).

### MGMT promoter methylation, and IDH 1 mutation testing

MGMT Promoter Methylation, and IDH1-R132H mutation testing were performed as previously described [22–24].

### DNA extraction

DNA was extracted from formalin-fixed, paraffin-embedded GBM sample sections with a QIAamp DNA FFPE Tissue Kit (Qiagen, Hildan. Germany), according to the manufacturer's instructions. DNA Samples were quantified with PicoGreen® (Life Technologies, Camarillo, CA, USA).

### CNV analysis with droplet digital (dd) PCR

ddPCR was performed with the QX100 Droplet Digital PCR system (Bio-Rad Laboratories, Hemel Hempstead, UK). The ddPCR reaction mixes in 22 μl DDW contained 11ul ddPCR supermix (Bio-Rad), 0.3 μM of each primer and probe and 10 to 30 ng of genomic DNA. Droplet generation and droplet reading for ddPCR were carried out with Bio-Rad reagents. The thermal cycling profile was 95°C:10′00′′ followed by 40 cycles of (95°C:0′15′′; 60°C:1′00′′). Analysis of the ddPCR data was performed with the Q×100 analysis software CNV mode (version 1.2.9.0), and relied on two probes, one specific to the AR labeled with FAM fluorescent signal and an internal reference Ribonuclease P (Rnase P) gene, with a single copy per haplotype that was assayed on the HEX channel. Each experiment was performed twice and in triplicate, with two sets of primers for AR (AR1 and AR2, as indicated below):

AR1-F:AGTGCCTGTTGGAGACAAGA;

AR1-R: ACCACCATGACGCAGAAGAG

AR1-probe (FAM): TCAAACGTATGTCCCTGT CGATCTCA

AR2-F:GGGCACAGAGACCTGGAAA;

AR2-R: CCTGACACCCTGATAACTACTCA

AR2-Probe (FAM): TCCATGTGCTAACCCATA TCCTGGC

Rnase-P-F:AGATTTGGACCTGCGAGCG;

Rnase-P-R:GAGCGGCTGTCTCCACAAGT

Rnasep-P-Probe (HEX): TTCTGACCTGAAGG CTCTGCGCG.

### RNA extraction, cDNA preparation and qPCR

Total RNA was isolated from snap frozen gliomas and cell cultures with TRI Reagent® (Sigma-Aldrich, Rehovot, Israel), according to the manufacturer's instructions. Control RNAs were obtained from a commercial mix pooled from 23 donors (mean age, 68 years; 13 men and 10 women) (FirstChoice® Human Brain Reference Total RNA, Thermo Fisher Scientific Inc., Waltham, MA, USA).

cDNA was produced from 0.2ug total RNA with a qScript cDNA Synthesis Kit (Quanta Biosciences, Gaithersburg, MD, USA), according to the manufacturer's instructions.

Real-time PCR amplification and relative quantification were analyzed with StepOne real time RT PCR (Life Technologies). The reaction mix included 1μl cDNA, and 300 nmol/l of each of the following primers (Syntezza, Jerusalem, Israel):

AR(1)-F:ACCGAGGAGCTTTCCAGAATC,

AR(1)-R:AGGCTCTGGGACGCAACCT;

AR(2)-F: CTCCTTTGCAGCCTTGCTCTCT,

AR(2)-R: ACGTGTAAGTTGCGGAAGCCA

HPRT-F:GATGGTCAAGGTCGCAAGC,

HPRT-R:ATATCCTACAACAAACTTGTCT GGAA;

TBP-1-F:CCACTCACAGACTCTCACAAC,

TBP-1-R:CTGCGGTACAATCCCAGAACT

and 5μl of SYBR green mix (Perfecta Syber Green Fast Mix ROX, Quanta Biosciences) in a total 10μl volume. The fold changes of the target mRNAs were normalized to HPRT and TBP1. Then the fold changes of each mRNA were calculated based on the ratio between the analyzed tumor/cell line sample and normal tissue, as indicated. The experiment was repeated three times in triplicate and the results are presented as the mean ± SD.

### mRNA expression of AR splice variants

AR variant 7 (AR3) was analyzed with quantitative (q)PCR, as described above, with the following primer pair (adopted from Rong Hu et al.) [8]:

AR-7-F:CCATCTTGTCGTCTTCGGAAATGTT ATGAAGC

AR-7-R:TTTGAATGAGGCAAGTCAGCCTTTCT.

The resulting 125bp fragments were also electrophoresed on 3.5% metaphor, visualized with ethidium bromide and validated according to Sanger sequencing with the forward primer.

### Western blot analysis

Western blotting was performed as previously described [25] with minor modifications. Briefly, tissue samples or cell line pellets were homogenized in 500 ul RIPA Lysis and Extraction Buffer (Thermo Fisher Scientific Inc.) supplemented with protease inhibitors (Thermo Fisher Scientific Inc.). The protein concentration was determined according to the Bradford protein assay (Bio-Rad, Richmond, CA, USA). Tissue/cell line lysates containing 100ug protein were separated on 4%-20% Tris-Glycine SDS-PAGE gel (Thermo Fisher Scientific Inc) and assessed according to Western blot analysis, and sequential probing with a polyclonal antibody against AR (N20, 1:200 dilution); a monoclonal anti- GAPDH (0411, diluted 1:10000) (Santa Cruz Biotechnologies, Santa Cruz, CA, USA,) or anti-β-Actin (AC-74 diluted 1:5000) (Sigma-Aldrich), as indicated, and with the relevant secondary horseradish peroxidase-conjugated antibody (Santa Cruz Biotechnologies).

### Cell culture

Cell lines A172, U87MG and T98G (glioblastoma) were obtained from the American Type Culture Collection (Manassas, VA, USA). A172 and U87MG cells were cultured in Dulbecco's modified Eagle's medium (DMEM) supplemented with 4 mmol/L L-glutamine, 100 units/ml penicillin,100 μg/ml streptomycin and with either (as indicated) 10% of full FBS or charcoal/dextran-treated (stripped) FBS (Biological Industries, Beit Haemek, Israel), to avoid the influence of FBS-derived steroid hormone on AR. The T98G cells were cultured in Eagle's minimum essential medium supplemented with L-glutamine, penicillin and streptomycin and 10% charcoal/dextran-treated (stripped) FBS. Two glioma-initiating cell (GICs) lines, ZH-161 and ZH-305, were kindly provided by Prof. Michael Weller from the Department of Neurology at the University Hospital Zurich, Switzerland and maintained as described [13, 14]. Briefly, cells were cultured in Neurobasal Medium (Gibco; Thermo Fisher Scientific, Inc., Waltham, MA, USA)) supplemented with B-27 (20 μl/ml) and glutamax (10 μl/ml), fibroblast growth factor (FGF)-2, epidermal growth factor (EGF) (20 ng/ml each (Peprotech, Rocky Hill, PA, USA) and heparin (32 IE/ml; Sigma-Aldrich). All cells were maintained in a humidified incubator at 37°C in 5% CO^2^.

### Treatment with AR inhibitors *in vitro*

A total 1×10^3^ cells were plated in triplicate in 24-well plates, (Thermo Fisher Scientific Inc) and allowed to attach overnight. The growth medium was replaced with medium containing physiological amounts (10nM) of DHT (Sigma-Aldrich) [This concentration had no effect on cell viability in any of the cell lines compared with that of cells treated with vehicle (0.15% ETOH) (see Figure 2) and the indicated concentrations of AR inhibitors, enzalutamide (A2S technologies, Yavne, Israel) or bicalutamide (Sigma-Aldrich), for 48h and/or 72, as indicated.

### siRNA transfection

A total 2 × 10^4^ A172, U87 and T98G cells were plated in triplicate in 24-well plates. After 24hrs the cells were transfected with 1.2 nM of the indicated siRNA by using INTERFERIN (Polyplus; New York, NY, USA) transfection reagent according to the manufacturer's instructions. AR knockdown was performed with ON-TARGETplus Human AR (367) siRNA (Dharmacon; Lafayette, CO, USA). The ON-TARGETplus Non-targeting Pool siRNA (Dharmacon ) served as control. mRNA levels of full length AR were quantified after 24h with Real Time RT-PCR by using two sets of primers (AR1 and AR2) as described above and cell survival was measured after 72h with the aforementioned method.

### Cell survival analysis

The crystal violet binding assay was used to measure the viability of A172, U87MG and T98G standard cell lines, as described previously [26]. Briefly, 0.5% crystal violet (Sigma-Aldrich) was added to each well following fixation of the cells with 4% paraformaldehyde solution in phosphate-buffered-saline (PBS) (Sigma-Aldrich). The dye was solubilized with 10% acetic acid (Sigma-Aldrich) and absorbance was read at 590 nm in a DTX 880 multimode detector microplate reader (Beckman Coulter, Nyon, Switzerland). The average absorbance value of the control was considered 100% and the treated sample percentages were calculated according to the average absorbance of the treated samples/the average absorbance of the control.

The viability of the GIC lines was assessed as previously described [27]. Briefly, GICs were seeded in neurobasal medium (Gibco), and incubated for 24 hrs. Then exposed to DHT and enzalutamide for 72hrs at the dosages described above and the 3-(4,5-dimethylthiazol-2-yl)-2,5-diphenyltetrazolium bromide (MTT) method was used according to the manufacturer's instructions.

### Cell cycle analysis with propidium iodide (PI) DNA staining

A172, U87 and T98G glioma cell lines were treated with vehicle (0.15% ETOH), 10nm of DHT with or without 40 or 80uM enzalutanide as indicated above for 48hrs for A172 and U87MG and 72 hrs for T98G. Then the cells were harvested with trypsin and washed with PBS. This was followed by fixation overnight at -20°C in cold 80% ethanol. After washing the cells with PBS and treatment with 50 μl of a 100 μg/ml solution of RNAse, 200 μl PI (from a 50 μg/ml stock solution) were added. PI fluorescence intensity was captured with flow cytometry by using the FL2 channel and 488nM laser excitation.

### Immunofluorescence staining of cleaved caspase 3

A172, U87MG and T98G glioma cell lines were treated as indicated above for 72 hrs. Then the cells were fixed in 4% paraformaldehyde for 10 min at RT and permeabilized with cold methanol for 20 min. The cells were then washed twice with PBS-T and incubated in blocking solution (3% BSA, 0.1% Tween in PBS) for 30 min. The cells were incubated with primary antibody (Asp175) (1:50) against Cleaved Caspase-3, (Cell Signaling Technology, Danvers, MA. USA) for 60 min at 37°C and Alexa fluor 488 conjugate secondary antibody (1:200) anti-rabbit IgG (cat No. A21206, Invitrogen, Waltham, Ma., USA) for 60 minutes at RT. The cell nuclei were stained with DAPI (D9542, Sigma-Aldrich) and the cells were visualized and captured with an EVOS® Digital Microscope at 200 x magnification.

### *In vivo* U87MG human glioblastoma xenografts

Ethical statement: The study was carried out in accordance with the recommendations in the Guide for the Care and Use of Laboratory Animals of the National Institutes of Health. The protocol was approved by the Committee on the Ethics of Animal Experiments of the Hebrew University Medical School (Permit Number: MD-16-14864-5). To minimize the suffering of the animals, injections of tumor cells were performed under light anesthesia (ketamine + xylazine, 100 and 5 mg/kg body weight, respectively). The animals were monitored twice weekly for tumor size and body weight. Upon termination of the experiments, the animals were euthanized by exposure to excess CO2.

A total 5×10^6^ glioblastoma cells (U87MG) were inoculated subcutaneously in the interscapular area of 26 female athymic nude mice. Tumor growth was monitored with the aid of a hand-held Vernier caliper twice weekly. Tumor volume was estimated according to the following equation: width^2^×length/2 (see ref. [28]. On day 7, when the tumors reached an average volume of about 50 mm^3^, the mice were randomly assigned to two treatment groups, based on the caliper measurements. All the mice were treated three time weekly by oral gavage with 20 mg/kg enzalutamide (Astellas Pharma Inc. Tokyo, Japan) (n=8) or vehicle (220mg/kg caprylocaproyl polyoxylglycerides in saline) (n=18). The enzalutamide dosage for the *in vivo* experiment was calculated according to the FDA guidelines for scaling of drug doses [dosage-converting tables from humans to mice can be found in [29]], and preclinical trials with enzalutamide in [30]. The mice were sacrificed when their tumors reached a volume of 800–1000 mm^3^, as requested by the local animal committee, in order to minimize the animals’ suffering.

### Statistical analysis

The statistical significance between the studied groups vs the control groups for each experiment was calculated according to Student's 2-tailed *t* test.

## SUPPLEMENTARY MATERIALS FIGURES AND TABLES


