# Supplementary material for: Androgen receptor: a potential therapeutic target for glioblastoma
Source: Oncotarget. 2018 Apr 13;9(28):19980–93. doi: 10.18632/oncotarget.25007 (PMC5929440; doi:10.18632/oncotarget.25007)
Supplement: Supplementary file 1 [file oncotarget-09-19980-s001.pdf]

Androgen receptor: a potential therapeutic target for glioblastoma

SUPPLEMENTARY MATERIALS

OncoScan analysis

To obtain genome-wide copy number and loss-of-heterozygosity (LOH) profiles from FFPE tumor samples, 80 ng FFPE-derived DNA were subjected to an OncoScan FFPE Express 2.0 | Affymetrix array (Affymetrix Inc., Santa Clara, CA, USA). The assay was performed according to the manufacturer's instructions. The data were analyzed with Nexus 6 Copy Number™ (Biodiscovery, El Segundo, CA, USA).

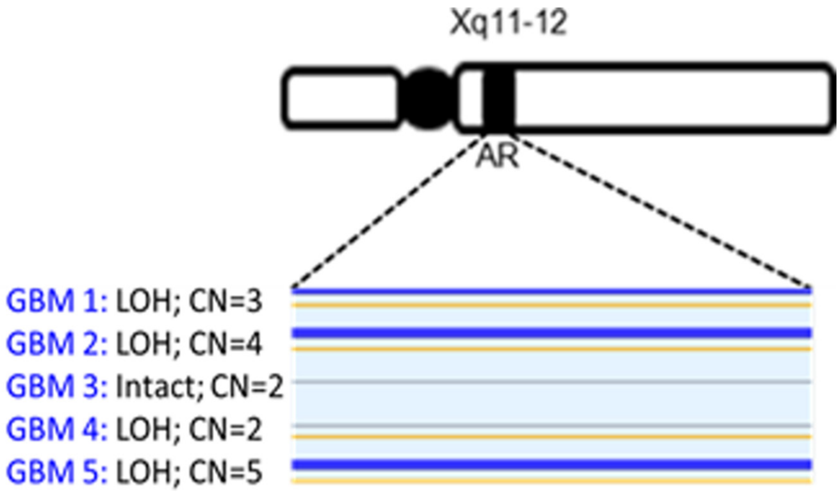

Supplementary Figure 1: genome-wide CNV and LOH array of DNA extracted from five samples of five GBM from women. AR chromosomal region captured with Nexus Express Software. The yellow area represents LOH; the blue area - CN gain. The state of the whole X chromosome may be seen to the left of each figure.

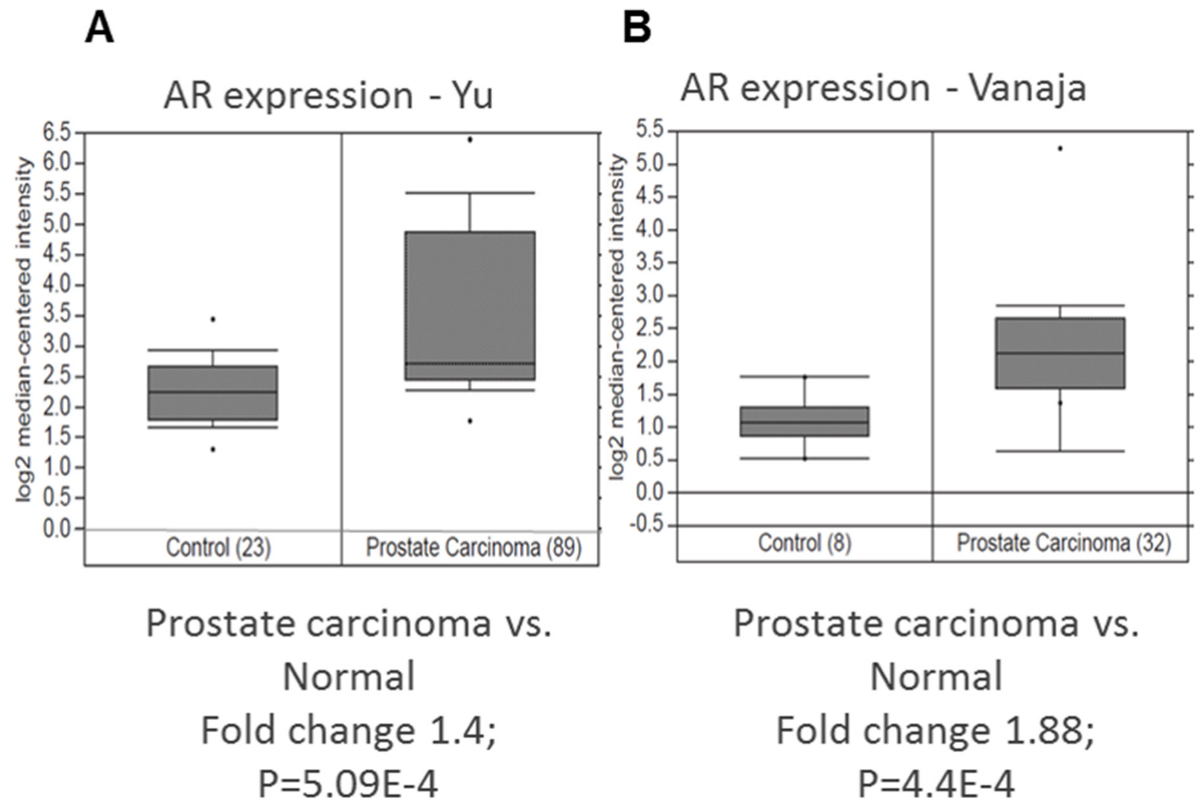

Supplementary Figure 2: (A-B) Oncomine™ analysis of AR-RNA amplification of two datasets of prostate cancer Yu (A) and Vanaja cohorts (B).

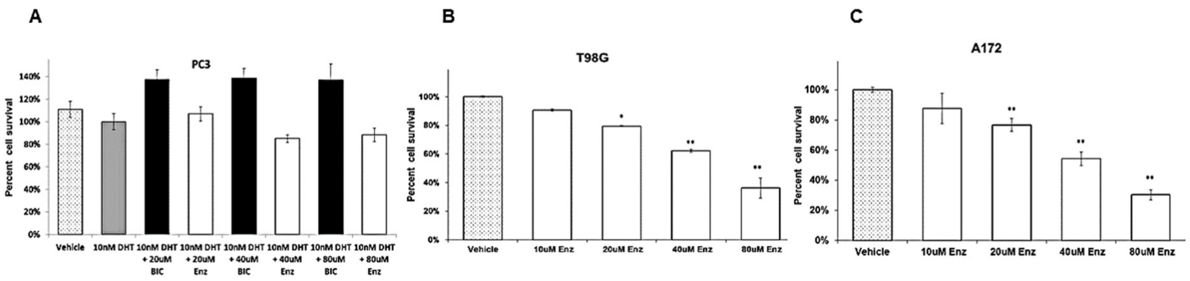

Supplementary Figure 3: The effect of AR antagonists on cells survival. (A) PC3 cells were treated with DHT alone (grey bars) or in combination with 20 μM, 40 μM or 80 μM of bicalutamide (BIC, blacks bars) or enzalutamide (ENZ, white bars) (X axis), for 72 Hrs. as indicated. Cell viability was determined by crystal violet assay and expressed as the percentage of cells treated with DHT (Y axis).**(B-C)** cells were treated with 10 μM 20 μM, 40 μM or 80 μM of ENZ in cell culture medium supplemented with full serum without addition of DHT. Cell viability was determined by crystal violet assay and expressed as the percentage of cells treated with Vehicel (Y axis). (B)T98G cell line. (C) A172 cell line.
